# Deciphering phenotyping, DNA barcoding, and RNA secondary structure predictions in eggplant wild relatives provide insights for their future breeding strategies

**DOI:** 10.1038/s41598-023-40797-z

**Published:** 2023-08-24

**Authors:** Sansuta Mohanty, Bandana Kumari Mishra, Madhumita Dasgupta, Gobinda Chandra Acharya, Satyapriya Singh, Ponnam Naresh, Shyamlal Bhue, Anshuman Dixit, Arup Sarkar, Manas Ranjan Sahoo

**Affiliations:** 1 Central Horticultural Experiment Station, ICAR–Indian Institute of Horticultural Research, Bhubaneswar, Odisha 751019 India; 2 Trident Academy of Creative Technology, Bhubaneswar, Odisha 751024 India; 3 ICAR Research Complex for Northeastern Hill Region, Manipur Centre, Imphal, Manipur 795004 India; 4 ICAR–Indian Institute of Horticultural Research, Bengaluru, 560089 Karnataka India; 5 Institute of Life Sciences, Bhubaneswar, Odisha 751024 India; 6 Present Address: Department of Molecular Biology and Biotechnology, Institute of Agricultural Sciences (IAS), Siksha O Anusandhan, Deemed to be University, Bhubaneswar, Odisha 751003 India

**Keywords:** Biotechnology, Plant sciences

## Abstract

Eggplant or aubergine (*Solanum melongena* L.) and its wild cousins, comprising 13 clades with 1500 species, have an unprecedented demand across the globe. Cultivated eggplant has a narrow molecular diversity that hinders eggplant breeding advancements. Wild eggplants need resurgent attention to broaden eggplant breeding resources. In this study, we emphasized phenotypic and genotypic discriminations among 13 eggplant species deploying chloroplast–plastid (*Kim matK*) and nuclear (*ITS2*) short gene sequences (400–800 bp) at DNA barcode region followed by *ITS2* secondary structure predictions. The identification efficiency at the *Kim matK* region was higher (99–100%) than in the *ITS2* region (80–90%). The eggplant species showed 13 unique secondary structures with a central ring with various helical orientations. Principal component analysis (PCoA) provides the descriptor–wise phenotypic clustering, which is essential for trait–specific breeding. Groups I and IV are categorized under scarlet complexes *S. aethiopicum*, *S. trilobatum*, and *S. melongena* (wild and cultivated). Group II represented the gboma clade (*S. macrocarpon*, *S. wrightii*, *S. sisymbriifolium*, and *S. aculeatissimum*), and group III includes *S. mammosum*, and *S. torvum* with unique fruit shape and size. The present study would be helpful in genetic discrimination, biodiversity conservation, and the safe utilization of wild eggplants.

## Introduction

Eggplant and its wild relatives (*Solanum spp.*) are major genera in the family *Solanaceae,* contributing over 1500 plant species worldwide^1^. The genus *Solanum* is subdivided into 13 clades comprising 450 species^2^. Eggplant or aubergine (*Solanum melongena* L.) and some of its wild cousins are native to the Indian subcontinent having an unprecedented demand across the globe^3^. The gboma (*Solanum macrocarpon* L.) and scarlet eggplant (*Solanum aethiopicum* L.) of old–world origin are also cultivated as minor vegetable crops all over the world^4^. Production of eggplant reached over 51 million tonnes from an area of 1.8 million ha worldwide^5^. India is the second–largest producer of eggplants after China, accounting for global production of 13.4 million tonnes^6^. Transforming from the ancient world to commercial cultivation, wild relatives of eggplant are often considered the prime source of genetic materials in trait–specific breeding programs. The crop wild relatives (CWR) are hostile species with inherent tolerance towards various abiotic and biotic stresses such as drought, salinity, heavy metals, high temperature, disease, and pest menace. Thus, CWRs became the breeder’s first choice while augmenting breeding strategies for crop improvement^7^.

Adaptation of wild relatives of eggplant in India is as old as its civilization and was reported to be used as Unani medicines since time immemorial. Wild eggplants contribute antimicrobial, insecticidal, antiviral, and anti–carcinogenic properties^8^. Numerous species of wild edible eggplant contain high phenolic compounds with crypto chlorogenic acid and neochlorogenic acid associated with low lipids and high levels of antioxidants^9^. Antioxidants in the eggplant skin have a cytotoxic effect in activating apoptosis and building cells resistant to premature aging^10^. Some wild eggplant peel contains nasunin anthocyanin, a potent antioxidant and a potential pharmaceutical factor for several human diseases like diabetes, cancer, and cardiovascular diseases^11^. Apart from many nutraceutical properties and health benefits, the wild and cultivated eggplant is an essential source of dietary fibre, carbohydrates, potassium, manganese, phenols, glycol–alkaloids, and vitamins B, C, and K^12^. The significant role of eggplant wild relatives in food and nutritional security is well recognized.

Despite being an important genetic resource for eggplant breeding, research advancement on taxonomic identification, protection, genetic conservation, and improvement of wild eggplant relatives are limited^13^. Wild eggplant landraces are often neglected like an orphan crop with a restricted distribution to the cultivated species, threatening their extinction^14^. It has also been observed that the cultivated eggplant has a narrow molecular diversity than the wild ones, a major hindrance to eggplant breeding advancements^15^. Wild relatives need resurgent attention to obtain advanced breeding resources through classical and molecular approaches. Hence, urgent attention is required to identify, characterize, and conserve eggplant biological diversity as a genetic reservoir for future breeding strategies. Accurately identifying wild edible eggplants is remarkably convenient for their safe utilization, environmental protection, and prevention of perceived biodiversity loss. With the acceptance of the global exploitation of this crop, the taxonomic and DNA–based molecular identification of the wild eggplant relatives holds a promising involvement in providing a greener technique. Phenological and molecular characterization of wild relatives offers an understanding of the desirable traits leading to biotic and abiotic stress management^13^. Species identification using morphological descriptors and PCR–based markers is time and labor–intensive. In recent years, DNA barcoding evolved as an efficient and reliable tool to describe the genetic relationship between plant species and their wild relatives faster.

DNA barcoding discriminates the species using a standardized short gene sequence of 400–800 bp derived from a conserved genome region^16^. It can be applied in species identification the same way as commercial products are identified with black strips that encode the Universal Product Code^17^. This technique is well established in animals but is complex and poses a challenge in plants as they require multiple loci, and distinguishing closely related species is difficult. No universal barcode candidate has been identified to determine the plant species^18^. DNA barcoding aims to construct barcode sequence libraries of all known species that can easily be accessible to identify or match the known and unknown species. DNA barcoding eliminates the errors presumed in traditional taxonomic identification due to morphological mutagenesis and genotypic and phenotypic variability^19^. Following modern advancement, DNA barcode confirms high throughput species discrimination from a small amount of tissue at any plant growth period^17^.

DNA barcodes targeting several candidate gene regions, such as mitochondrial, plastid, and nucleus, are well adopted in plant species discrimination studies^20^. Ribulose-1,5-bisphosphate carboxylase–oxygenase (*rbcL*), maturase K (*MatK, XF/5R*) or *Kim matK* (*3F Kim and 1R Kim*), *trnH–psbA*, and internal transcribed spacer (*ITS*) are the standard DNA barcodes used in plant species discrimination^21^. The nuclear *ITS* region, located at 45S ribosomal RNA (rRNA), subdivided into *ITS1* and *ITS2* regions, was recommended for species identification in most plant species due to the higher rate of PCR amplification^22^. *Kim matK* (*3F Kim and 1R Kim*), the most promising and well–conserved plastid coding regions in the chloroplast, are the most reliable barcode primers for species identification among land plants^23^. The Consortium of Barcode of Life (CBOL-Plant working group, 2009)^24^ suggested a combination of plastid (*Matk*/*Kim matK*) and nuclear region (*ITS*) as a potent barcode tool to examine plant species discrimination^20^.

RNA secondary structure predictions at conserved *ITS* rRNA region is a key ribosomal structure that predicts the function of rRNAs and tRNAs^25^. Computationally predicted RNA structure represents the native RNA folding status of an organism that sheds light on novel RNA regulatory mechanisms^26^. RNA secondary structure prediction is an advanced tool for species discrimination as it restricts sequencing error and eliminates pseudogene footprints^27^.

Many researchers across the globe have been working on genetic discrimination and taxonomic identification of wild, underutilized species using morphological indicators, PCR–based biomolecular characterization, and DNA barcode markers. Morphological descriptors discriminate the plant species following phylogeny, which requires in–depth knowledge of plant characteristics^28^ involving a taxonomist. However, DNA barcoding and RNA secondary structure predictions authenticate the species delamination through molecular phylogeny^29^. Morphological indicators and DNA barcode–based signature molecular events have been used to discriminate species in various plants^29,30^.

The present study involves the identification of wild eggplant relatives following phenological characterization and molecular documentation using DNA barcode markers at the chloroplast region (*Kim matK*) and nuclear region (*ITS*). We have also aimed to predict RNA secondary structures to understand the genetic discriminations among the wild eggplants that substantiates the penological phylogeny at the molecular level. The result of this study would enable accurate identification of wild eggplant relatives for augmenting trait–specific genetic improvement of eggplant.

## Results

### Phenotypic variations among the eggplant wild relatives

**Figure 1:**
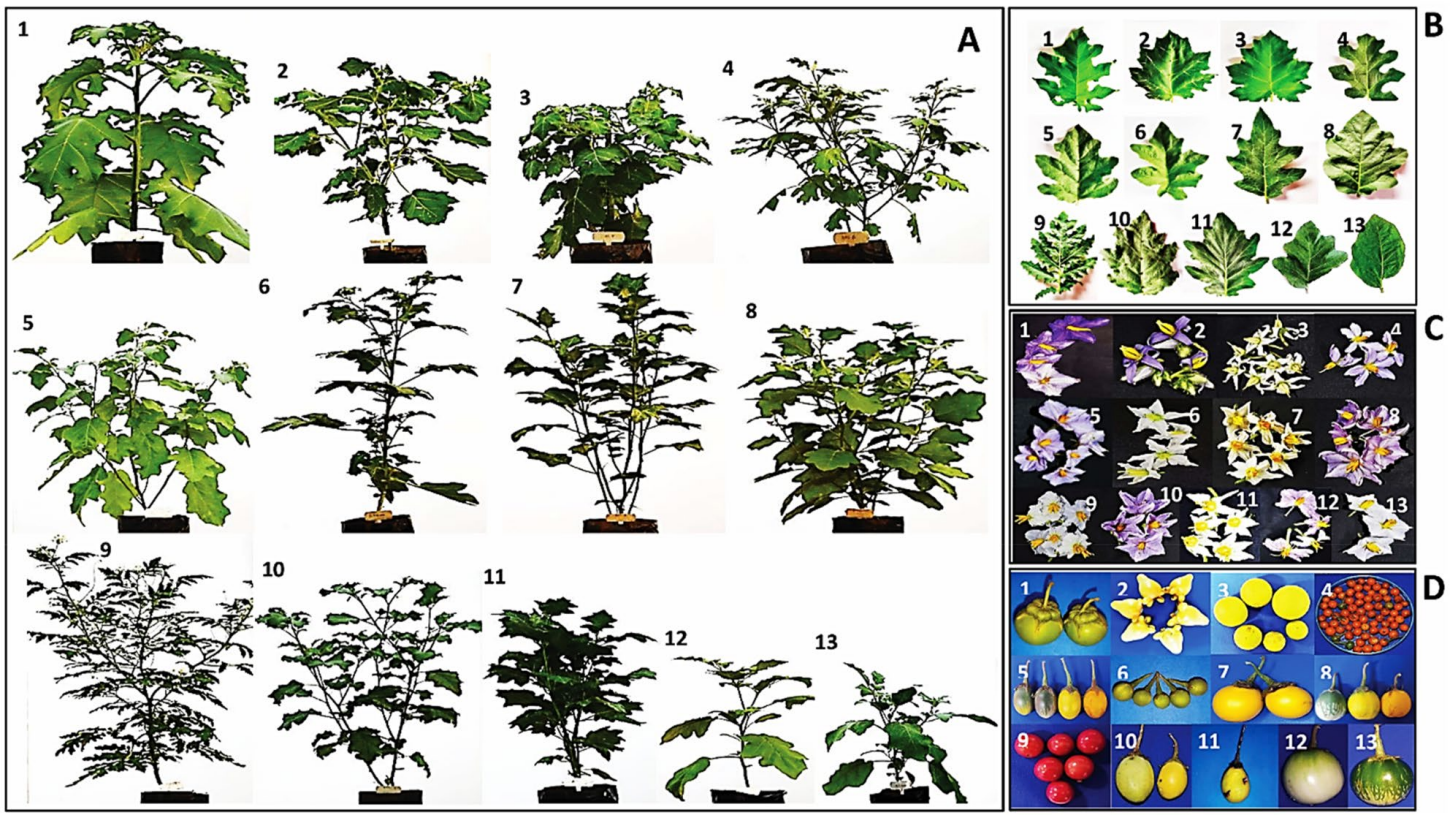
(**A**–**D**) Plant phenotypic features, (**A**) Plant growth habit, (**B**) Leaf characters, (**C**) Flower morphology, and (**D**) fruit characters of 13 eggplant wild relatives [1. CHB WEP–1, 2. CHB WEP–2, 3. CHB–WEP–3, 4. CHB–WEP–4, 5. CHB–WEP–5, 6. CHB–WEP–6, 7. CHB–WEP–7, 8. CHB–WEP–8, 9. CHB–WEP–9, 10. CHB–WEP–10, 11. CHB–WEP–11, 12. CHB–WEP–12, 13. CHB–WEP–13].

Significant variations (*P* ≤ *0.01*) were observed among the 33 out of 40 phenotypic descriptors (Supplementary Table 1) recorded for the 13 eggplants and their wild relatives (CHB WEP 1–13; Table 1). The tested genotypes have shown no significant differences in the seven conventional descriptors, such as stem anthocyanin, anthocyanin intensity, fruit calyx color, leaf margin, blade color, vein color, and blistering. Figure 1A–D depicts the morphological discriminations such as plant phenotype, leaf features, floral morphology, and fruit characters, respectively.

**Table 1:** List of the eggplant and its wild relatives used for the genetic discrimination study.

Reference ID	Voucher No	Local name	Scientific name	Accession
IIHR–CHB–WEP–1	CHB WEP–1	Giant star potato tree	*S. wrightii*	Wild
IIHR–CHB–WEP–2	CHB WEP–2	Cow’s udder/Nipple fruit	*S. mammosum*	Wild
IIHR–CHB–WEP–3	CHB–WEP–3	CHB–5	*S. aculeatissimum*	Wild
IIHR–CHB–WEP–4	CHB–WEP–4	BRS–6	*S. trilobatum*	Wild
IIHR–CHB–WEP–5	CHB–WEP–5	BRS–20	*S. melongena*	Wild
IIHR–CHB–WEP–6	CHB–WEP–6	Pea eggplant/Devil’s fig	*S. torvum*	Wild
IIHR–CHB–WEP–7	CHB–WEP–7	BRS–3	*S. marcocarpon*	Wild
IIHR–CHB–WEP–8	CHB–WEP–8	Bitter brinjal	*S. aethiopicum*	Wild
IIHR–CHB–WEP–9	CHB–WEP–9	Sticky nightshade	*S. sisymbriifolium*	Wild
IIHR–CHB–WEP–10	CHB–WEP–10	Brinjal–16	*S. melongena*	Wild
IIHR–CHB–WEP–11	CHB–WEP–11	BRS–2	*S. aethiopicum*	Wild
IIHR–CHB–WEP–12	CHB–WEP–12	Brinjal	*S. melongena*	Cultivated
IIHR–CHB–WEP–13	CHB–WEP–13	Brinjal	*S. melongena*	Cultivated

### Phenotypic distance clustering among the eggplant wild relatives

**Figure 2:**
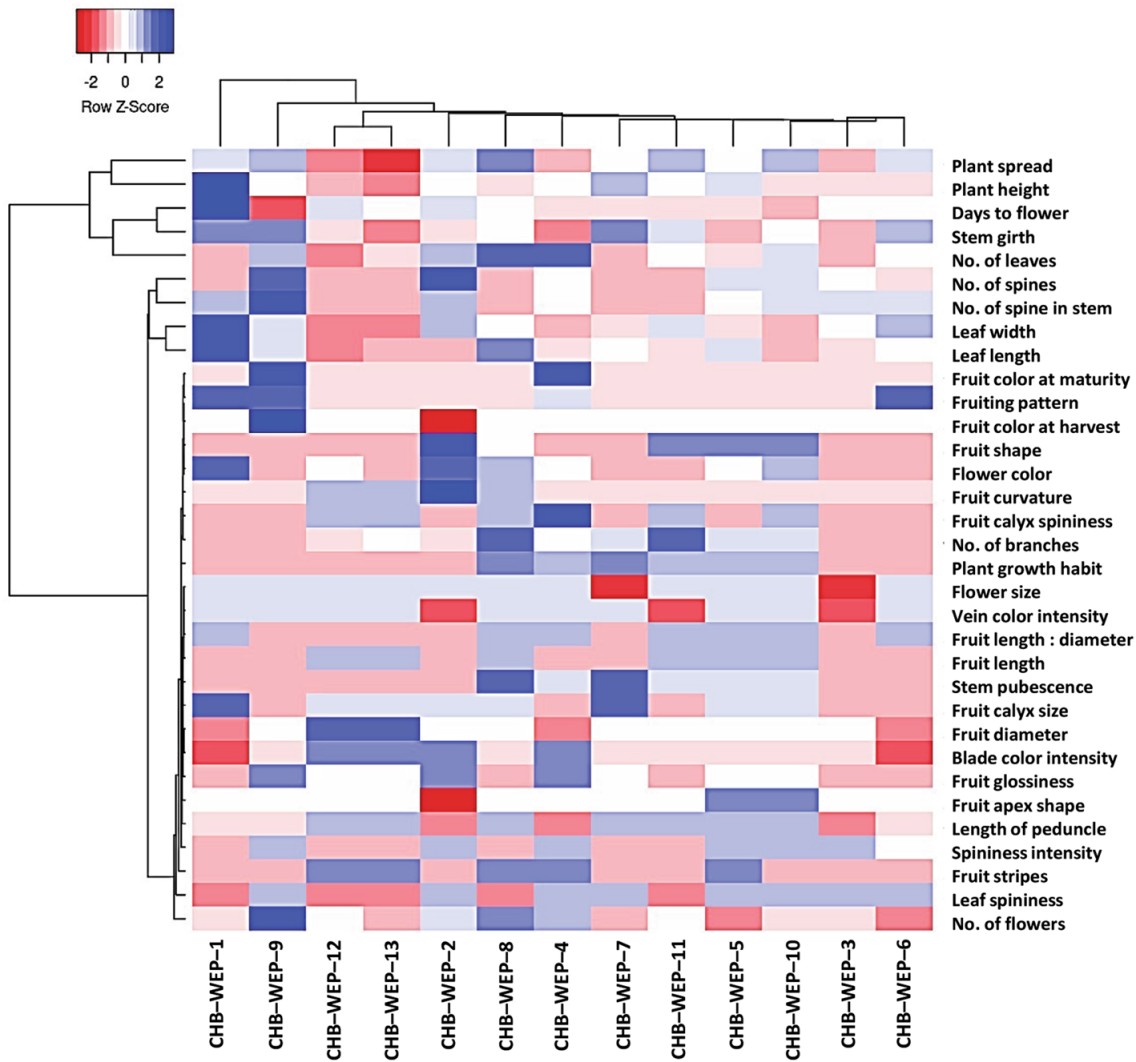
Heat map depicting the phenotypic association among 13 eggplant wild relatives.

Figure 2 represents the phenotypic distance among the 13 eggplant species based on the morphological descriptors. The eggplant species were divided into four major groups. Group I (*S. wrightii*, CHB–WEP–1) and group II (*S. sisymbriifolium*, CHB–WEP–9) exhibited unique morphotypes compared to other species. Group III includes two cultivated eggplant species of *S. melongena* (CHB–WEP–12 and CHB–WEP–13) with similar morphological features. Other species such as *S. mammosum* (CHB–WEP–2), *S. aculeatissimum* (CHB–WEP–3), *S. trilobatum* (CHB–WEP–4), *S. melongena*–wild type (CHB–WEP–5 and CHB–WEP–10), *S. torvum* (CHB–WEP–6), *S. macrocarpon* (CHB–WEP–7)*, S. aethiopicum* (CHB–WEP–8 and CHB–WEP–11) are categorized under group with a comparatively narrow phenotypic distance. Group IV was further divided into three subgroups with significant phenotypic variation among *S. mammosum* (Subgroup 1), *S. aculeatissimum,* and *S. torvum* (Subgroup 2), and Subgroup 3 includes *S. aethiopicum*, *S. trilobatum*, *S. macrocarpon*, and two wild types of *S. melongena*.

The phenotypic tree in the heatmap also describes the critical conventional features that predominantly discriminate the eggplant species (Fig. 2). The phenotypic descriptors were grouped into three major clusters. Group I includes plant spread, plant height, days to flower, stem girth, and the number of leaves which showed higher variability among the tested eggplant species. Group II comprises the number of spines in leaves and stems, leaf width, and girth, However, Group III comprises 24 conventional phenotypic characters, further grouped into three Subgroups (Fig. 2).

Fruit characters such as fruit color at maturity, fruiting pattern, fruit color at harvest, fruit shape, fruit curvature, fruit calyx spine, and flower color in Subgroup1 of Group III signifies less variation among the eggplant species, which may be used for stringent selection of the unique species. On the other hand, fruit glossiness, fruit ápex shape, length of the peduncle, spines intensity, fruit stripes, leaf–spine, and flower numbers in Subgroup 2 showed moderate variation. Subgroup 3 (number of branches, plant growth habit, flower size, vein color intensity, fruit length: diameter, fruit length, stem pubescence, fruit calyx size, fruit diameter, blade color intensity) showed a minimum impact on phenotypic discrimination among the eggplant species (Fig. 2).

### Principal component analysis

**Figure 3:**
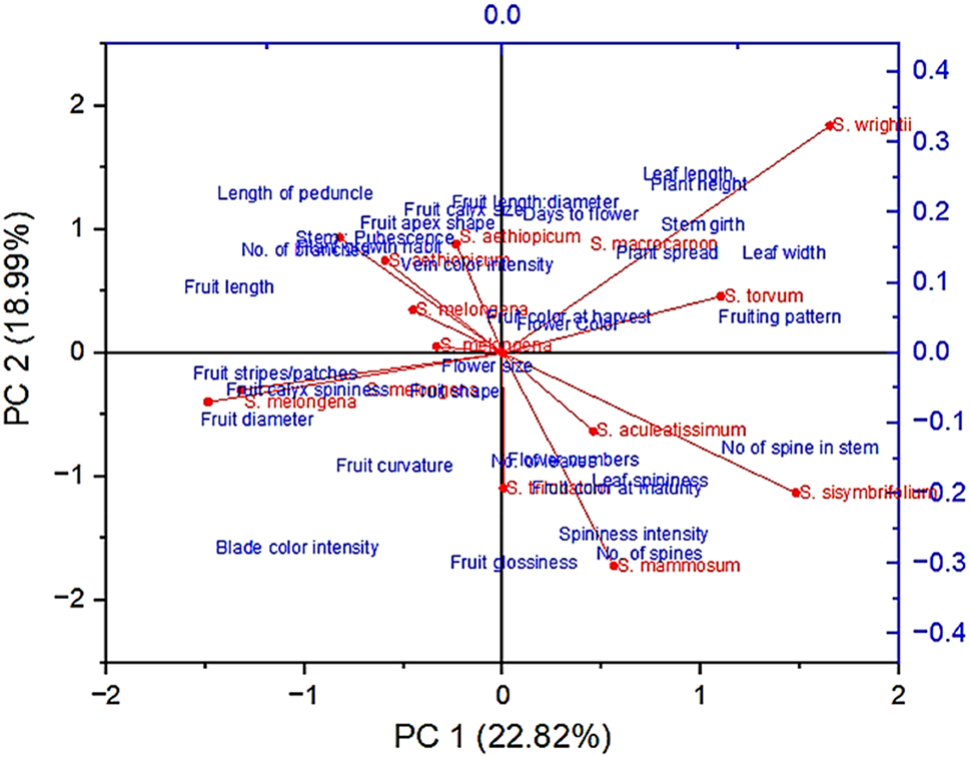
Principal component analysis (PCoA) depicting the trait–specific phenotypic association among 13 eggplant wild relatives.

Principal component analysis (PCoA; Fig. 3) represented the phenotypic descriptor–wise genotypic clustering, which validates the clusters obtained from the heat map. As per the PCoA result, CHB–WEP–1 (*S. wrightii*) and CHB–WEP–6 (*S. torvum*) differed from other species in terms of plant height, stem girth, plant spread, leaf length and width, fruiting pattern, and fruit and flower color. CHB–WEP–2, CHB–WEP–3, CHB–WEP–4, and CHB–WEP–9 exhibited spiny features in the leaf and stem and discriminated from other species based on the fruit color at maturity and the number of flowers. Two cultivated eggplant species (CHB–WEP–12, and CHB–WEP–13) were categorized in the same group with larger fruit shape, size, curvature, fruit stripes, and calyx spininess. The rest of the five eggplant species plotted in the fourth quadra varied among each other with nine morphological descriptors (Fig. 3).

### Correlation studies

**Figure 4:**
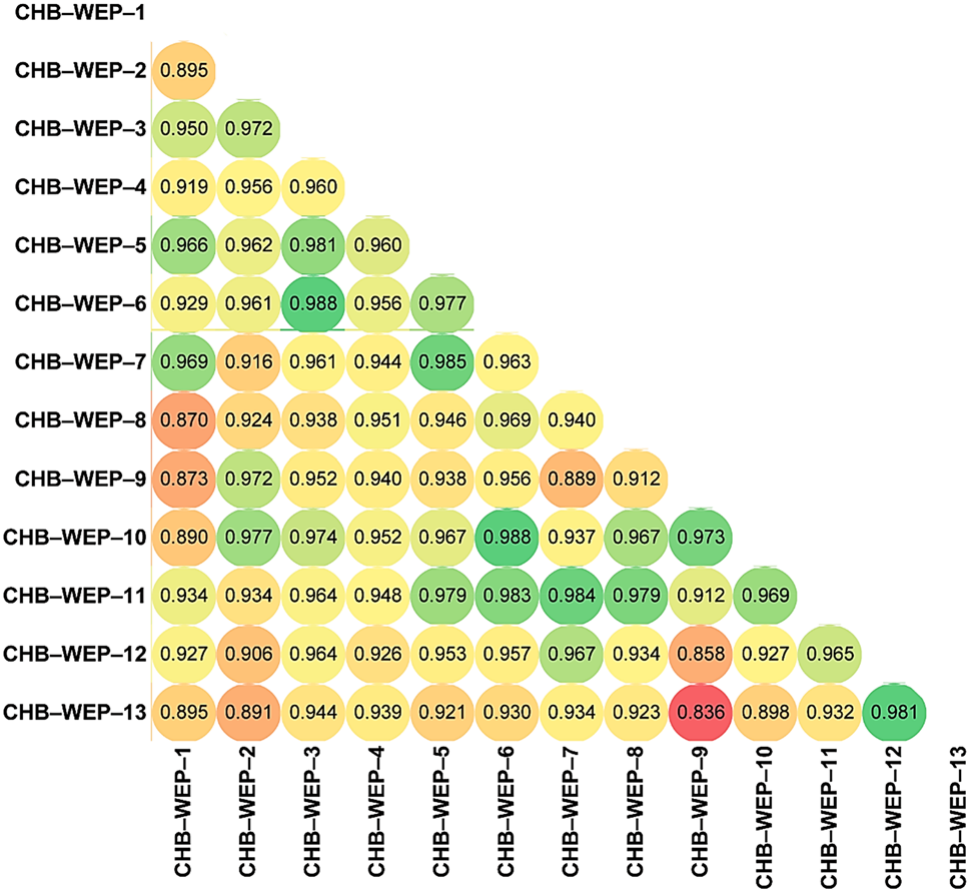
Correlation among the 13 eggplant wild relatives based on the phenotypic features. The threshold value at *P* ≤ *0.001* is r = 94.763.

Pearson’s correlation revealed phenotypic discriminations among the tested species at *P* ≤ *0.001* level of significance with a threshold value (r = 94.763) [Fig. 4]. The red color dots in Fig. 4 indicated the lowest, and the Green dots represented the highest correlation among the tested eggplant species. Based on the 33 phenotypic descriptors, CHB–WEP–11 (*S. aethiopicum*) possesses significant similarities with five species (two wild genotypes of *S. melongena*, *S. torvum*, *S. macrocarpon*, and *S. aethiopicum* (CHB–WEP–8). The wild *S. melongena* (CHB–WEP–10) resembled five different species (*S. mammosum*, *S. aculeatissimum*, *S. torvum*, *S. aethiopicum*, and *S. sisymbriifolium*) with similar morphological characteristics. The two cultivated species of *S. melongena* (CHB–WEP–12 and 13) showed the least morphological similarity to other species, indicating their narrow genetic base. There is a need for the incorporation of the wild genetic base to broaden the genetic trait among the cultivated species. The combined understanding of phenotypic phylogeny, principal component analysis, and correlation studies would be helpful in selecting the suitable species for augmenting trait–specific breeding strategies.

### Species discrimination using DNA barcoding

Table 2 represents the molecular identification of eggplant wild relatives using *Kim matK*, and *ITS2* barcode genes with 100% identity. Sequence characteristics of the candidate barcodes have been presented in Supplementary Table 2. The máximum sequence length was 856–1500 in *Kim matK* and 545–850 in *ITS2*. However, the aligned sequence length was achieved in the range of 463–812 and 295–460 for *Kim matK*, and *ITS2*, respectively (Supplementary Table 2). DNA barcoding significantly discriminated the tested eggplant species at chloroplast–plastid (*Kim matK,* ON623021–ON623033) and nuclear (*ITS2*, ON707266–ON707275, and ON968710–ON968712) regions. Multiple sequence alignments (MSA) using muscle algorithm obtained from the good quality sequences after trimming and filling the barcode gaps indicated a distinct genetic variability among the eggplant wild relatives at the species level. In our study, the identification efficiency at the *Kim matK* region was higher (99–100%) than that of the *ITS2* region (80–90%).

**Table 2:** Molecular identification of eggplant and its wild relatives using *Kim matK*, and *ITS2* barcode genes.

Biological reference number	Scientific name	*KimMatK*			*ITS2*		
		Accession number	Per cent identity	E value	Accession number	Per cent identity	E value
CHB–WEP–1	*S. wrightii*	ON623021	100%	0.0	ON707266	100%	0.0
CHB–WEP–2	*S. mammosum*	ON623022	100%	0.0	ON968710	100%	8 × 10^–173^
CHB–WEP–3	*S. aculeatissimum*	ON623023	100%	0.0	ON707267	100%	0.0
CHB–WEP–4	*S. trilobatum*	ON623024	100%	0.0	ON707268	100%	0.0
CHB–WEP–5	*S. melongena*	ON623025	100%	0.0	ON707269	100%	0.0
CHB–WEP–6	*S. torvum*	ON623026	100%	0.0	ON707270	100%	0.0
CHB–WEP–7	*S. marcocarpon*	ON623027	100%	0.0	ON968711	100%	0.0
CHB–WEP–8	*S. aethiopicum*	ON623028	100%	0.0	ON707271	100%	1 × 10^–150^
CHB–WEP–9	*S. sisymbriifolium*	ON623029	100%	0.0	ON707272	100%	2 × 10^–163^
CHB–WEP–10	*S. melongena*	ON623030	100%	0.0	ON707273	100%	0.0
CHB–WEP–11	*S. aethiopicum*	ON623031	100%	0.0	ON707274	100%	0.0
CHB–WEP–12	*S. melongena*	ON623032	100%	0.0	ON968712	100%	0.0
CHB–WEP–13	*S. melongena*	ON623033	100%	0.0	ON707275	100%	0.0

### Molecular phylogeny using máximum likelihood tree

**Figure 5:**
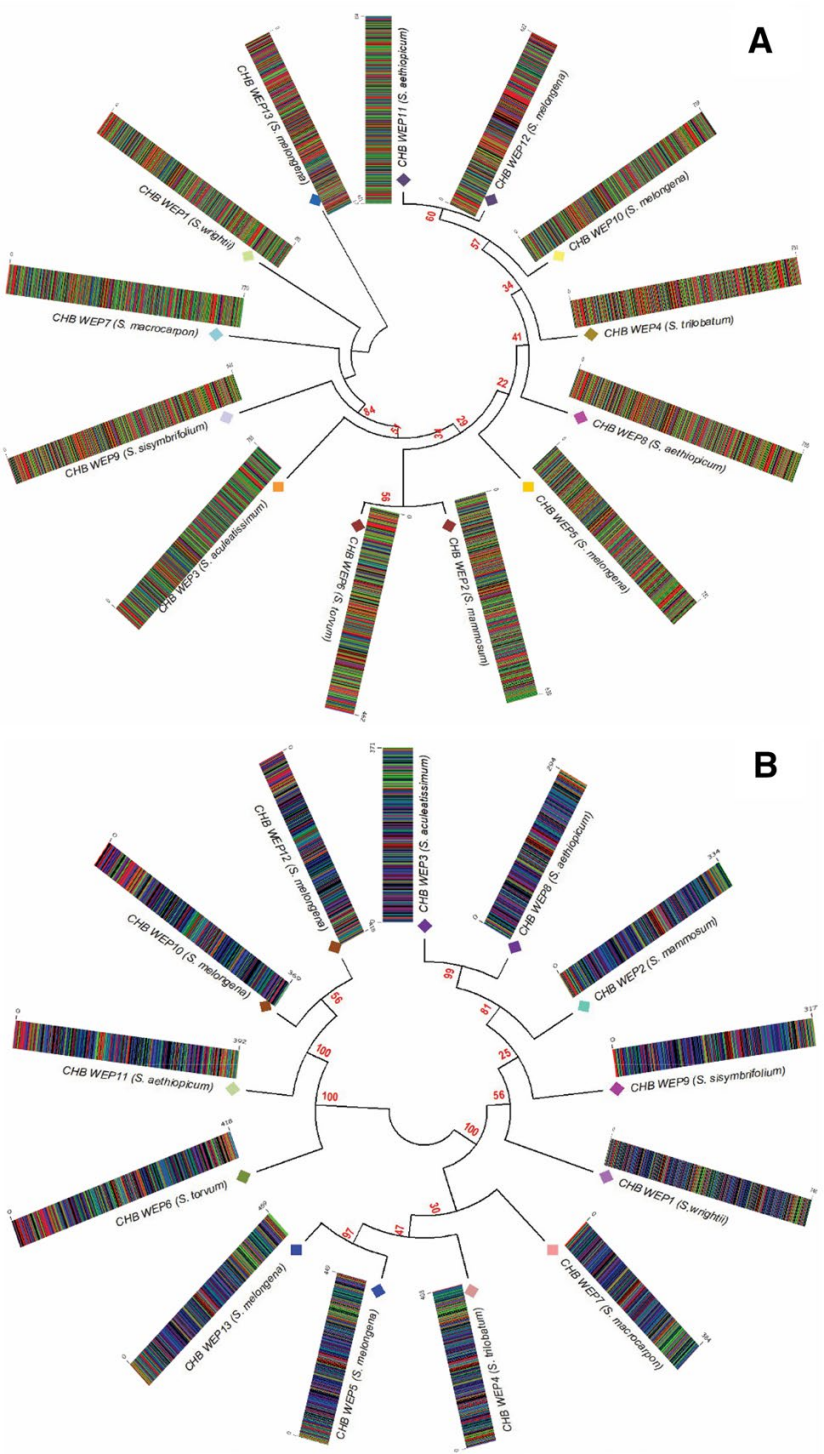
Maximum likelihood tree and DNA barcodes obtained from *Kim matK* (**A**) and *ITS2* (**B**) sequences depicting the relationship among 13 eggplant wild relatives. The bootstrap scores (1000 replicates) were shown (≥ 50%) for each branch**.**

Figure 5A,B depicted the phylogenetic relationships among the wild eggplant species and the barcodes obtained from *Kim matK*, and *ITS2* sequences, respectively. The phylogeny was established using a maximum likelihood tree (MLT) in a *K2P* model with bootstrap–1000. The MLTs distinctly categorized the 13 eggplant species into four major monophyletic groups. CHB–WEP–13, CHB–WEP–1, and CHB–WEP–2 were consistently categorized under Groups I, II, and III in the phylogeny at the *Kim matK*, and *ITS2* region. CHB–WEP–10, CHB–WEP–11, and CHB–WEP–12 appeared together in one clade (cluster IV). However, *ITS2* MLT confirmed the similarities of CHB–WEP–13 with CHB–WEP–4, 5, and 7. The MLT based on *Kim matK* locus data, CHB–WEP–1, showed similarities with CHB–WEP–3, 7, and 9. Groups I and IV are categorized under scarlet complexes *S. aethiopicum*, *S. trilobatum*, and *S. melongena* (wild and cultivated). Whereas Group II represented the gboma clade (*S. macrocarpon*, *S. wrightii*, *S. sisymbriifolium*, and *S. aculeatissimum*). However, the intermediate Group III includes *S. mammosum*, and *S. torvum* with unique features in fruit shape and size.

### *ITS2* secondary structure predictions

**Figure 6:**
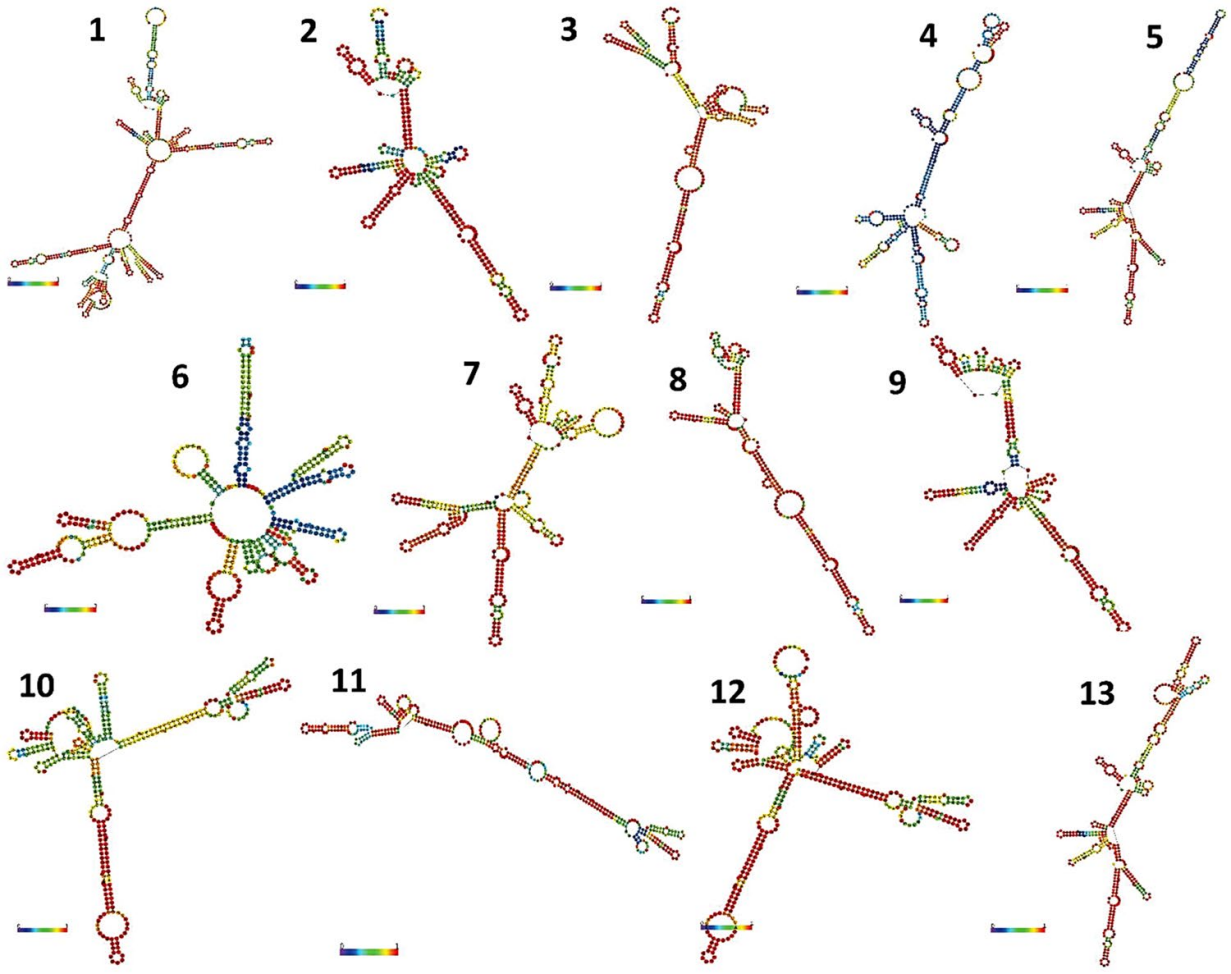
The predicted minimum free energy (MFE) secondary structures of *ITS2* region from 13 eggplants and its wild relatives (consensus structure), Conserved motif 5′–UGGU–3′ and U–U mismatch was detected [1. CHB WEP–1 (*S. wrightii*), 2. CHB WEP–2 (*S. mammosum*), 3. CHB–WEP–3 (*S. aculeatissimum*), 4. CHB–WEP–4 (*S. trilobatum*), 5. CHB–WEP–5 (*S. melongena*), 6. CHB–WEP–6 (*S. torvum*), 7. CHB–WEP–7 (*S. macrocarpon*), 8. CHB–WEP–8 (*S. aethiopicum*), 9. CHB–WEP–9 (*S. sisymbriifolium*), 10. CHB–WEP–10 (*S. melongena*), 11. CHB–WEP–11 (*S. aethiopicum*), 12. CHB–WEP–12 (*S. melongena*), 13. CHB–WEP–13 (*S. melongena*)].

We have predicted *ITS2* secondary structures for the 13 eggplant wild relatives (Fig. 6). The studied species showed 13 unique secondary structures with four similar helices, which implied the genetic variations among the species. Most species represented a central ring with various helical orientations regarding the loop number, position, size, and angle from the spiral. Helix I comprised of three species (*S. wrightii*, *S. macrocarpon*, and *S. mammosum*), helix II includes six eggplant genotypes. In contrast, helix III (*S. torvum*) and IV (*S. sisymbriifolium*) showed unique structures with multi–central rings. Helix V includes wild *S. melongena* (CHB–WEP–5) that predicted a unique but similar structure as predicted in cultivated *S. melongena* (CHB–WEP–13), which indicated their near–isogenic nature. The secondary structure prediction is important in the molecular breeding of eggplants interrogating wild relatives. The unique genetic sequences at the conserved nuclear region would also help to develop species–specific primers for the identification of wild eggplants at a faster pace.

## Discussion

### Phenotyping of eggplant wild relatives

Phenotyping using morphological descriptors is crucial for the preliminary identification and selection of genotypes for breeding and crop improvement^31^. In the present study, thirteen diverse eggplant species were phenotypically discriminated using the forty morphological descriptors illustrating a close relationship among the eggplant wild relatives. Among all the significant heritable descriptors, plant height, stem features, leaf characters, and fruit characters (fruit shape, size, and color) could be considered reliable traits to distinguish eggplant diversity phenotypically^32^. Leaf structural descriptors, such as the spiny features in wild eggplants, served as a potential basis for morphological discrimination among the wild and cultivated ones. The species closer to the wild eggplants support the assumption of interspecific hybrids revealing the general observation of allelic uniformity^13^. Overlapping phenotypic features in the same genus *Solanum* correlates with the genetic interrelationship among the wild and cultivated eggplant gene pool. Following the hypothesis of the gene pool notion, Howard et al.^32^ suggested that although the wild progenitors and the cultivars possess some morphological similarities, they might differ at the genotypic/species level, which needs to be confirmed at the molecular level.

In the eggplant improvement program, *S. torvum* and *S. mammosum* are often used as a primary gene pool for interspecific hybridization against biotic and abiotic stress^33^. Similarly, introgression of *Solanum incanum* and *Solanum lichtensteinii* are accomplished for broadening eggplant genetic diversity^13^. In our study, PCoA suggests considering *S. wrightii*, *S. torvum*, and *S. macrocarpon* for trait–specific breeding for plant height, plant spread, stem girth, and fruiting pattern. *S. sisymbriifolium, S. mammosum, S. trilobatum*, and *S. aculeatissimum* are grouped under the leaf and stem spininess may be selected for breeding for spiny characters. *S. melongena,* and *S. aethiopicum* could be selected for breeding for better fruit characteristics. The morphological clustering in our study would help select suitable species for improving introgression breeding strategies to develop stress–tolerant eggplant species. The detailed analysis of morphological characters represents a powerful technique for analyzing the phenomic relationship among wild and cultivated eggplant species^34^. However, DNA–based molecular tools may be considered for species identification and gene bank conservation^35^.

### DNA barcoding of eggplant wild relatives

Various molecular markers are often used for the genetic characterization of plant species to identify quantitative and qualitative trait–specific loci. However, the accurate identification of a species is practically complicated using taxonomic or molecular characterization. In the present study, we have efficiently used the DNA barcode markers (*Kim matK*, and *ITS*) to accurately discriminate the species of the thirteen eggplant wild relatives at the molecular level. Specific candidate barcode markers such as *Kim matK* (chloroplast–plastid region) and *ITS* (nuclear region) were often deployed for species identification in many plants^32^. Consequently, molecular barcoding approaches can provide a tool to identify novel eggplant species–specifically. Using the advancement of DNA barcode, *ITS* and *Kim matK* barcode loci efficiently discriminate *Solanaceae* family at species level^36^. The genotypes with significant barcode gaps may be considered for inter- or intraspecific eggplant breeding strategies. The genetic information at a particular barcode location is suitable for enhancing eggplant breeding techniques. The genotypes with fewer DNA barcode sequence gaps could be chosen for breeding eggplants with specific traits.

### *ITS2* secondary structure predictions in eggplant wild relatives

The RNA secondary structure can be categorized based on three main criteria: minimum free energy, a technique based on statistical value, and evaluating the nucleotide sequence^37^. The RNA secondary model presumes that RNA folding occurs in a stable structure with the lowest free energy. RNA secondary structure prediction is a novel method to elucidate RNA folding in plant cell physiology. Few studies in plant RNA structure predictions, especially those of agriculturally important crops, have been attempted. However, a genome–wide RNA structure map has been inferred in vivo using *A. thaliana* seedlings^38^. Expanding the findings of such methods, we focused on advancement in understanding the outline and role of RNA structure in plants. The prediction accuracy by comparing and investigating a considerable figure of homologous RNA molecular sequences of different plant species is tricky in discriminating the variation using RNA secondary structure.

Identification based on the nuclear coding region of ribosomal subunit (28S and 5.8S coding region) using *ITS* primers is now a reliable tool for species–level specification^39^. The barcode–based molecular analysis of RNA secondary structure using *ITS* sequences for species evolution interferes with their target genetic loci. For correct discrimination of all the 13 genotypes of eggplant germplasm, additional information on the RNA folded model appears to be relevant in determining the divergence between all closely related eggplant variants. The complementarities accounting for the regions of the folded structure were found to be identical in domain base pairing, forming a core region by correlating it with some stem features^38,39^. The revealed order of predilection is maintained on the topology of RNA structure based on the inner loop, bulge variety loop, hairpin, and outer loop of all eggplant species. Hence, the studied relationship among the eggplant variants depends upon the prediction effect of the results of *ITS* sequence conservativeness in the preferred nuclear region. The species with closer barcode gaps represent the same clade suitable for inter or intra–specific eggplant breeding.

## Conclusions

CHB–WEP–1 (*S. wrightii*) and CHB–WEP–6 (*S. torvum*) exhibited unique plant characteristics such as plant height, stem girth, plant spread, and fruiting pattern. CHB–WEP–2 (*S. mammosum*), CHB–WEP–3 (*S. aculeatissimum*), CHB–WEP–4 (*S. trilobatum*), and CHB–WEP–9 (*S. sisymbriifolium*) exhibited spiny features, which could be considered for the trait–specific approaches with the cultivated *Solanum melongena* (CHB–WEP–12, and CHB–WEP–13) possessed better fruit shape, size, and curvature, and fruit stripes. The chloroplast–plastid gene *Kim matK* provided better species discrimination over the nuclear *ITS2*. The species discrimination was more prominent at DNA barcode regions, confirming the genotypic variations among the wild eggplant species. *Kim matK* could be used for the identification of new species or discrimination among large genetic populations. *ITS2* secondary structure predictions depict the unique genetic configuration at the conserved 5.8S nuclear region. Most species represented a central ring with various helical orientations regarding loop number, position, size, and angle from the spiral. This study shows the potential of DNA barcoding in discriminating eggplant wild relatives. Understanding the phenology and molecular phylogeny would be helpful for the selection of CWR for breeding strategies of eggplants.

## Materials and methods

### Plant materials and experimental conditions

Thirteen accessions of eggplant, including eleven wild and two cultivated species maintained at the Central Horticultural Experiment Station (CHES), Indian Council of Agricultural Research–Indian Institute of Horticultural Research (ICAR–IIHR), Bhubaneswar, India, with due approval of the competent authority following institutional guidelines and legislation, were used as the source materials for the present study. The station is located at a latitude of 20° 15′ N, a longitude of 85° 52′ E, and an altitude of 35 m above mean sea level.

Seeds of eggplant and its wild relatives were sown in pot trays containing cocopeat for germination under a naturally ventilated poly house (14 h photoperiod, 85–90% relative humidity, and temperature of 30/25 ºC day/night). Six weeks old seedlings were transplanted to the polyethene pots (30 × 30 × 30 cm) containing garden soil, sand, and farm yard manure (1:1:1) in the polyhouse. The plants were maintained as per the recommended package of practice for eggplant. The experiment was designed with 13 genotypes and five replications in a completely randomized design (CRD). The leaf voucher specimens (CHB WEP 1–13; Table 1) of the eggplant and its wild relatives were deposited in the herbarium at ICAR–IIHR–CHES, Bhubaneswar, India, which were used as biological reference material (BRM) in the present study.

### Morphological characterization

Morphological descriptors such as plant phenotypic features, leaf phenology, floral morphology, and fruit characters were recorded as per the distinctness, uniformity, and stability (DUS) guidelines for eggplant as recommended by the protection of plant variety and farmers’ rights authority (PPV&FRA)^40^, New Delhi, India. Data were analyzed using analysis of variances (ANOVA). Principal component analysis (PCoA), and heat map were illustrated using GraphPad Prism 9 (GraphPad Software, San Diego, CA, USA). The 13 eggplant species were characterized using 40 morphological descriptors at the whole plant level (Supplementary Table 1).

### Genomic DNA isolation and quantification

Total genomic DNA (gDNA) was isolated from the fresh juvenile leaf tissues of the 13 wild and cultivated eggplants using GCC–WLN plant gDNA extraction kit (GSure® Plant Mini Kit with WLN Buffer, GCC Biotech Pvt. Ltd., Kolkata, India) by following manufacturer’s protocol. The isolated gDNAs were quantified using a nanodrop spectrophotometer (Eppendorf, Hamburg, Germany) and checked on 0.8% agarose gel electrophoresis (Tarson, Kolkata, India). Total gDNA concentration adjusted to 50 ng µL^–1^ was used for PCR amplification with different barcode primers^24^.

### Primer selection and PCR amplification

DNA barcode primers for the chloroplast–plastid genome (*Kim matK*) and nuclear gene (*ITS2*) were synthesized at M/S Bioserve Biotechnologies India Pvt. Ltd., Hyderabad, India. The details of the barcode primer sequences (5’ to 3’) are, *Kim matK* (3F_*Kim matK*: CGTACAGTACTTTTGTGTTTACGAG; and 1R_*Kim matK*: ACCCAGTCCATCTGGAAATCTTGG) and *ITS2* (*ITS*–S2F: ATGCGATACTTGGTGTGAATTATAGAAT; and *ITS*–S3R: GACGCTTCTCCAGACTACAAT). For each chloroplast and nuclear marker, PCR amplification was performed in a volume of 25 µL, containing 50 ng of gDNA (1 µL) as a template, 12.5 µL 2 × PCR master mix (GCC Biotech Pvt. Ltd., Kolkata, India), primers (10 pM, 1 µL each of forward and reverse primers), and 9.5 µL Milli–Q water. All PCR amplifications were performed in the thermal cycler (Eppendorf, Hamburg, Germany) following denaturation of 5 min at 95 °C, 40 cycles of 1 min at 95 °C, 1 min at 55 °C of annealing, 1 min at 72 °C and a final extension of 10 min at 72 °C. The PCR products were purified using a PCR Purification Kit (GCC Biotech Pvt. Ltd., Kolkata, India) following the manufacturer’s instructions. The PCR–purified fragments were visualized in 1.5% agarose TAE gels, and the gel images were taken in the E–Box gel documentation system (Vilber, Eberhardzell, Germany).

### Sequencing and bioinformatics data analyses

The purified PCR products were sequenced using Sanger sequencing (ABI Genetic Analyzer 3730, 48 capillaries, 50 cm, ABI, Massachusetts, USA) at M/S Bioserve Biotechnologies India Pvt. Ltd., Hyderabad, and the sequences were viewed in FinchTV v1.4.0. Phylogenetic analysis of the 13 eggplants was carried out by the homology search of the obtained sequences using NCBI Basic Local Alignment Search Tool (BLAST, http://blast.ncbi.nlm.nih.gov) to identify the highest similarity of the eggplant accessions within the GenBank database^41^. Before submission of the sequences in NCBI, the analyzed forward and reverse sequences (*Kim matK* and *ITS*) were edited, trimmed, and contig formation was done using SnapGene v 5.3 (https://www.snapgene.com/). The nucleotides were BLAST, and the selection of the species was made based on the maximum similarity score, per cent identity (above 80%) and lowest E value after significant sequence alignment. The barcode gaps were manually edited in a pairwise alignment view using BLAST^42^. To obtain their respective accession numbers, the acquired *Kim matK* and *ITS* barcode sequences of each eggplant genotype were submitted to the BlankIt submission portal (https://submit.ncbi.nlm.nih.gov/subs/genbank/) and Genbank (https://submit.ncbi.nlm.nih.gov/) submission portal, respectively^43^. Around ten closest matches of the sequences were aligned with the query sequences by using Cluster Omega, and the resulting alignments were used to construct the phylogenetic tree by the neighbor–joining method. Multiple sequence alignment was run with all obtained sequences in the “muscle algorithm” using the neighbor–joining cluster method in MEGA11 software (Molecular Evolutionary Genetic Analysis; ClustalW v10.1.8; https://www.megasoftware.net)^44^. Two Neighbor–Joining trees were constructed by selecting phylogeny reconstruction with 1000 “*Bootstrap phylogeny”* test method and “*kimura–2–parameter”* substitution model (*d–transitions*) in MEGA software^45^. Phylogenetic relationships and evolutionary distance were studied using the minimum evolution method of *Kim matK*, and *ITS2* sequences. The maximum likelihood tree was estimated using MEGAX software considering the transitional and transversional nucleotide substitution. DNA barcodes were generated using Bio–Rad DNA barcode generator (http://biorad-ads.com/DNABarcodeWeb).

### RNA secondary structure prediction using ITS2 primer

RNA secondary structure represents the list of nucleotide bases paired by hydrogen bonding within its nucleotide sequence, and these base pairs form the scaffold driving the folding of RNA two– and three–dimensional structures. The knowledge of the RNA secondary structure is essential for modelling RNA structures and understanding their functional mechanism^43^. Target RNA structure is an important consideration in the design of small interfering RNAs and antisense DNA oligonucleotides. In the present study, the secondary structure of different eggplant genotypes was predicted using the DNA nucleotide sequences from *ITS–S2F* and *ITS–S3R* primers using *RNAfold* WebServer v2.4.18 (http://rna.tbi.univie.ac.at/cgi–bin/RNAWebSuite/RNAfold.cgi).

### Supplementary Information

Supplementary Tables.

## Data Availability

The data is available online (NCBI ID provided). However, all the data will be made available on request from the corresponding author (M.R.S: manas.sahoo@icar.gov.in).

## References

[1] Frodin DG (2004). History and concepts of big plant genera. Taxon.

[2] Knapp S, Vorontsova MS, Prohens J (2013). Wild relatives of the eggplant (*Solanum*
*melongena* L.: Solanaceae): New understanding of species names in a complex group. PLoS ONE.

[3] Polignano G, Uggenti P, Bisignano V, Gatta CD (2010). Genetic divergence analysis in eggplant (*Solanum*
*melongena* L.) and allied species. Genet. Resour. Crop Evol..

[4] Daunay MC, Hazra P, Peter KV, Hazra P (2012). Eggplant. Handbook of Vegetables.

[5] Tegally A, Jaufeerally-Fakim Y, Dulloo ME (2019). Molecular characterization of *Solanum*
*melongena* L. and the crop wild relatives, *S*. *violaceum* Ortega and *S*. *torvum* Sw., using phylogenetic/DNA barcoding markers. Genet. Resour. Crop Evol..

[6] Taher D, Solberg S, Prohens J, Chou Y, Rakha M, Wu T (2017). World Vegetable center eggplant collection: Origin, composition, seed dissemination and utilization in breeding. Front. Plant Sci..

[7] Devi YI, Sahoo MR, Mandal J, Dasgupta M, Prakash N (2020). Correlations between antioxidative enzyme activities and resistance to *Phytophthora* leaf blight in taro. J. Crop Improv..

[8] Hoque ME, Kashpia TP (2018). Molecular characterization and DNA fingerprinting of some local eggplant genotypes and its wild relatives. Int. J. Agric. Environ. Biotechnol..

[9] Petra S, Boulekbache-Makhlouf L, Pellati F, Ceslova L (2019). Monitoring of chlorogenic acid and antioxidant capacity of *Solanum*
*melongena* L. (Eggplant) under different heat and storage treatments. Antioxidants.

[10] Seraj H, Afshari F, Hashemi ZS, Timajchi M, Olamafar E, Ghotbi L (2017). Effect of eggplant skin in the process of apoptosis in cancer cells. STEM Fellow. J..

[11] Matsubara K, Kaneyuki T, Miyake T, Mori M (2005). Antiangiogenic activity of nasunin, an antioxidant anthocyanin, in eggplant peels. J. Agric. Food Chem..

[12] Naeem MY, Ugur S (2019). Nutritional content and health benefits of Eggplant. Turk. J. Food Sci. Technol..

[13] Kaushik P, Prohens J, Vilanova S, Gramazio P, Plazas M (2016). Phenotyping of eggplant wild relatives and interspecific hybrids with conventional and phenomics descriptors provides insight for their potential utilization in breeding. Front. Plant Sci..

[14] Syfert MM, Castaneda-Alvarez NP, Khoury CK, Sarkinen T, Sosa CC, Achicanoy HA, Bernau V, Prohens J, Daunay M-C, Knapp S (2016). Crop wild relatives of the brinjal eggplant (*Solanum melongena*): Poorly represented in genebanks and many species at risk of extinction. Am. J. Bot..

[15] Meyer RS, Karol KG, Little DP, Nee MH, Litt A (2012). Phylogeographic relationships among Asian eggplants and new perspectives on eggplant domestication. Mol. Phylogenet. Evol..

[16] Ford CS, Ayres KL, Toomey N, Haider N, Stahl LV, Kelly LJ, Wikstrom N, Hollingsworth PM, Duff RJ, Hoot SB, Cowan RS, Chase MW, Wilkinson MJ (2009). Selection of candidate coding DNA barcoding regions for use on land plants. Bot. J. Linn. Soc..

[17] Hartvig I, Czako M, Kjær ED, Nielsen LR, Theilade I (2015). The use of DNA barcoding in identification and conservation of rosewood (*Dalbergia* spp.). PLoS ONE.

[18] Mosa KA, Gairola S, Jamdade R, El-Keblawy A, Al Shaer KI, Al Harthi EK, Shabana HA, Mahmoud T (2019). The promise of molecular and genomic techniques for biodiversity research and DNA barcoding of the Arabian Peninsula Flora. Front. Plant Sci..

[19] Viglietti G, Galla G, Porceddu A, Barcaccia G, Curk F, Luro F, Scarpa GM (2019). Karyological analysis and DNA barcoding of *Pompia*
*Citron*: A first step toward the identification of its relatives. Plants.

[20] Han S, Sebastin R, Wang X, Lee KJ, Cho G-T, Hyun DY, Chung J-W (2021). Identification of *Vicia* Species native to South Korea using molecular and morphological characteristics. Front. Plant Sci..

[21] Kress WJ (2017). Plant DNA barcodes: Applications today and in the future. J. Syst. Evol..

[22] Chen S, Yao H, Han J, Liu C, Song J, Shi L (2010). Validation of the *ITS2* region as a novel DNA barcode for identifying medicinal plant species. PLoS ONE.

[23] Li Y, Gao L-M, Poudel RC, Li D-Z, Forrest A (2011). High universality of *matK* primers for barcoding gymnosperms. J. Syst. Evol..

[24] CBOL Plant Working Group (2009). A DNA barcode for land plants. Proc. Natl. Acad. Sci. USA.

[25] Gawronski P, Palac A, Scharff LB (2020). Secondary structure of chloroplast mRNAs *in vivo* and *in vitro*. Plants.

[26] Yang X, Yang M, Deng H, Ding Y (2018). New era of studying RNA secondary structure and its influence on gene regulation in plants. Front. Plant Sci..

[27] Rampersad SN (2014). *ITS1*, *5.8S *and *ITS2* secondary structure modelling for intra–specific differentiation among species of the Colletotrichum gloeosporioides sensu lato species complex. Springer Plus.

[28] Scotland RW, Olmstead RG, Bennett JR (2003). Phylogeny reconstruction: The role of morphology. Syst. Biol..

[29] Martinez-Arce A, De Jesus-Navarrete A, Leasi F (2020). DNA barcoding for delimitation of putative Mexican marine nematodes species. Diversity.

[30] Mohamed AH, Omar AA, Attya AM, Elashtokhy MMA, Zayed EM, Rizk RM (2021). Morphological and molecular characterization of some Egyptian six-rowed barley (*Hordeum*
*vulgare* L.). Plants.

[31] Portis E, Cericola F, Barchi L, Toppino L, Acciarri N, Pulcini L (2015). Association mapping for fruit, plant and leaf morphology traits in eggplant. Plos ONE.

[32] Howard C, Flather CH, Stephens PA (2020). A global assessment of the drivers of threatened terrestrial species richness. Nat. Commun..

[33] Rotino GL, Sala T, Toppino L (2013). Eggplant. Alien Gene Transfer Crop Plants.

[34] Feng Y, Sun R, Chen M, Liu C, Wang Q (2018). Simulation of the morphological structures of electrospun membranes. J. Appl. Poly. Sci..

[35] Singh A, Singh M, Singh R, Kumar S, Kalloo G (2006). Genetic diversity within the genus *Solanum* (*Solanaceae*) as revealed by RAPD markers. Curr. Sci..

[36] Rosario LH (2019). DNA barcoding of the Solanaceae family in Puerto Rico including endangered and endemic species. J. Am. Soc. Hort. Sci..

[37] Lu W, Cao Y, Wu H, Ding Y, Song Z, Zhang Y, Fu Q, Li H (2021). Research on RNA secondary structure predicting via bidirectional recurrent neural network. BMC Bioinform..

[38] Ding Y, Tang Y, Kwok CK, Zhang Y, Bevilacqua PC, Assmann SM (2014). *In vivo* genome–wide profiling of RNA secondary structure reveals novel regulatory features. Nature.

[39] Prasad PK, Tandon V, Biswal DK, Goswami LM, Chatterjee A (2009). Use of sequence motifs as barcodes and secondary structures of internal transcribed spacer 2 (*ITS2*, rDNA) for identification of the Indian liver fluke, Fasciola (Trematoda: Fasciolidae). Bioinformation.

[40] PPV&FRA. *Guidelines for the Conduct of Test for Distinctiveness**, **Uniformity, and Stability on Brinjal/Eggplant*. https://plantauthority.gov.in/sites/default/files/fbrinjal.pdf (2009).

[41] Premalatha K, Kalra A (2013). Molecular phylogenetic identification of endophytic fungi isolated from resinous and healthy wood of *Aquilaria **malaccensis*, a red listed and highly exploited medicinal tree. Fungal Ecol..

[42] Acharya GC, Mohanty S, Dasgupta M, Sahu S, Singh S, Koundinya AVV, Kumari M, Naresh P, Sahoo MR (2022). Molecular phylogeny, DNA barcoding, and *ITS2* secondary structure predictions in the medicinally important *Eryngium* genotypes of east coast region of India. Genes.

[43] Devi MP, Dasgupta M, Mohanty S, Sharma SK, Hegde V, Roy SS, Renadevan R, Kumar KB, Patel HK, Sahoo MR (2022). DNA barcoding and ITS2 secondary structure predictions in Taro (*Colocasia*
*esculenta* L. Schott) from the North Eastern Hill Region of India. Genes.

[44] Al-Juhani WS, Khalik KNL (2021). Identification and molecular study of medicinal *Plectranthus* species (Lamiaceae) from Saudi Arabia using plastid DNA regions and *ITS2* of the nrDNA gene. J. King Saud Univ..

[45] Kumar S, Stecher G, Li M, Knyaz C, Tamura K (2018). MEGA X: Molecular evolutionary genetics analysis across computing platforms. Mol. Biol. Evol..

