# Supplementary material for: Deciphering phenotyping, DNA barcoding, and RNA secondary structure predictions in eggplant wild relatives provide insights for their future breeding strategies
Source: Sci Rep. 2023 Aug 24;13:13829. doi: 10.1038/s41598-023-40797-z (PMC10449851; doi:10.1038/s41598-023-40797-z)
Supplement: Supplementary file 1 — Supplementary Tables. [file 41598_2023_40797_MOESM1_ESM.docx]

Supplementary Material

Deciphering Phenotyping, DNA Barcoding, and RNA Secondary Structure Predictions in Eggplant Wild Relatives Provide Insights for Their Future Breeding Strategies

**Sansuta Mohanty^1,†^, Bandana Kumari Mishra^1,2,†^, Madhumita Dasgupta^3,†^, Gobinda Chandra Acharya^1^, Satyapriya Singh^1^, Ponnam Naresh^4^, Shyamlal Bhue^5^, Anshuman Dixit^5^, Arup Sarkar^2^ and Manas Ranjan Sahoo^1,*^**

***Correspondence:** Corresponding Author: manas.sahoo@icar.gov.in

**Supplementary Table 1.** Plant phenotypic features, leaf phenology, floral morphology, and fruit characters are used for the characterization of the eggplant and its wild relatives.

| **Sl. No.** | | **Characteristics** | **States** | **Note** |
| --- | --- | --- | --- | --- |
| **Whole Plant** | | | | |
| 1 | Plant growth habit | | Erect  Semi spreading  Spreading  Horizontal | 1  5  7  9 |
| 2 | Plant height | | Very short (<30 cm)  Short (30–60 cm)  Medium (61–100 cm)  Tall (101–150 cm) | 1  3  5  7 |
| 3 | Plant spread | | Narrow (<50 cm)  Medium (50–100 cm)  Broad (>100 cm) | 3  5  7 |
| 4 | No. of branches | | Low (<3)  Medium (3–5)  High (>5) | 1  3  5 |
| **Stem Characters** | | | | |
| 5 | Stem anthocyanin coloration | | Absent  Present | 1  9 |
| 6 | Stem intensity of anthocyanin coloration | | Weak  Medium  Strong  Very strong | 3  5  7  9 |
| 7 | Stem pubescence | | Erect  Semi spreading  Spreading  Horizontal | 1  5  7  9 |
| 8 | Stem diameter (girth) | | Small (<1 cm)  Medium (1–5 cm)  Large (>5 cm) | 3  5  7 |
| 9 | No. of spines in stem | | Weak (<5)  Medium (5–10)  Strong (>10) | 3  5  7 |
| **Leaf Characteristics** | | | | |
| 10 | Leaf length | | Small (<10 cm)  Medium (10–20 cm)  Strong (>20 cm) | 3  5  7 |
| 11 | Leaf width | | Small (<10 cm)  Medium (10–20 cm)  Strong (>20 cm) | 3  5  7 |
| 12 | Leaf margin | | Entire  Dentate  Sinuate | 1  3  5 |
| 13 | Leaf blistering | | Absent  Present | 1  9 |
| 14 | Leaf spininess | | Absent  Present | 1  9 |
| 15 | Intensity of spininess (leaf) | | Weak (<5)  Medium (5–10)  Strong (>10) | 3  5  7 |
| 16 | Leaf blade color | | Green  Purple | 1  2 |
| 17 | Leaf blade color intensity | | Light  Medium  Dark | 3  5  7 |
| 18 | Leaf vein color | | Green  Purple | 1  2 |
| 19 | Leaf vein color intensity | | Light  Medium  Dark | 3  5  7 |
| 20 | No. of leaves | | <5  5–10  >10 | 3  5  7 |
| **Floral Morphology** | | | | |
| 21 | Number of flowers (inflorescence) | | 1–3  >3 | 1  2 |
| 22 | Flower size | | Small  Medium  Large | 3  5  7 |
| 23 | Flower color | | Greenish  Light purple  Purple  Dark purple | 1  2  3  4 |
| 24 | Flowering time (days after seed sowing) | | Early (<60 days)  Medium (60–80 days)  Late (>80 days) | 3  5  7 |
| **Fruit Characteristic** | | | | |
| 25 | Fruit shape (General) | | Globular  Ovoid  Obovate  Pear shaped  Club shaped  Ellipsoid  Cylindrical | 1  2  3  4  5  6  7 |
| 26 | Fruit shape (Apex) | | Indented  Flattened  Rounded  Pointed | 1  2  3  4 |
| 27 | Fruit curvature (only for cylindrical types) | | Absent  Slight  Medium  Strong | 1  3  5  7 |
| 28 | Fruit skin color at Comercial harvesting | | White  Green  Purple | 1  2  3 |
| 29 | Fruit stripes/patches | | Absent  Present | 1  9 |
| 30 | Fruit glossiness at harvest maturity | | Weak  Medium  Strong | 3  5  7 |
| 31 | Fruit length | | Short (<10 cm)  Medium (10–20 cm)  Long (>20 cm) | 3  5  7 |
| 32 | Fruit diameter | | Small (<5 cm)  Medium (5–10 cm)  Large (>10 cm) | 3  5  7 |
| 33 | Fruit length : diameter | | Small (<1.0)  Medium (1.0–2.0)  Large (>2.0) | 3  5  7 |
| 34 | Fruit calyx color | | Green  Purple | 1  2 |
| 35 | Fruit calyx size | | Small  Medium  Large | 3  5  7 |
| 36 | Fruit calyx spineness | | Absent  Weak  Medium  Strong | 1  3  5  7 |
| 37 | No. of spines (fruit calyx) | | Weak (<5)  Medium (5–10)  Strong (>10) | 3  5  7 |
| 38 | Length of peduncle | | Short (<1 cm)  Medium (1–5 cm)  Long (>5 cm) | 3  5  7 |
| 39 | Fruiting pattern | | Solitary  Cluster  Mixed | 1  2  3 |
| 40 | Fruit color at maturity | | Yellow  Orange  Brown | 1  2  3 |

**Supplementary Table 2.** Sequence characteristics of candidate barcodes.

| **Voucher specimens** | ***Kim matK*** | | | | ***ITS2*** | | | | |
| --- | --- | --- | --- | --- | --- | --- | --- | --- | --- |
|  | **Accession Number** | **Total**  **score** | **Align**  **ment length** | **GC content** | | **Accession Number** | **Total score** | **Align**  **ment length** | **GC content** |
| CHB–WEP–1 | ON623021 | 1500 | 812 | 32.0 | | ON707266 | 749 | 405 | 69.2 |
| CHB–WEP–2 | ON623022 | 1182 | 640 | 34.1 | | ON968710 | 619 | 444 | 69.6 |
| CHB–WEP–3 | ON623023 | 1448 | 784 | 33.3 | | ON707267 | 688 | 372 | 65.1 |
| CHB–WEP–4 | ON623024 | 1389 | 752 | 32.7 | | ON707268 | 747 | 404 | 60.6 |
| CHB–WEP–5 | ON623025 | 1352 | 732 | 33.3 | | ON707269 | 832 | 450 | 65.6 |
| CHB–WEP–6 | ON623026 | 856 | 463 | 33.5 | | ON707270 | 774 | 419 | 59.7 |
| CHB–WEP–7 | ON623027 | 1424 | 771 | 33.2 | | ON968711 | 712 | 443 | 67.5 |
| CHB–WEP–8 | ON623028 | 1452 | 786 | 32.7 | | ON707266 | 545 | 295 | 68.8 |
| CHB–WEP–9 | ON623029 | 1482 | 802 | 33.3 | | ON707272 | 588 | 318 | 70.1 |
| CHB–WEP–10 | ON623030 | 1330 | 720 | 33.5 | | ON707267 | 684 | 449 | 68.6 |
| CHB–WEP–11 | ON623031 | 1478 | 800 | 32.9 | | ON707268 | 726 | 393 | 62.8 |
| CHB–WEP–12 | ON623032 | 1151 | 623 | 36.1 | | ON707269 | 774 | 419 | 65.2 |
| CHB–WEP–13 | ON623033 | 1242 | 672 | 32.7 | | ON707270 | 850 | 460 | 64.1 |
